# Ultra-high altitude environment triggers life-threatening gastrointestinal bleeding in undiagnosed Crohn's disease: a case report

**DOI:** 10.3389/fmed.2026.1770748

**Published:** 2026-04-08

**Authors:** Jing Li, Yelu Hao, Jie Zhou, Xiaoqin Ha, Zhibiao Cai

**Affiliations:** 1 Department of Clinical Pharmacology, The 940th Hospital of Joint Logistics Support Force of PLA, Lanzhou, Gansu, China; 2 Department of Neurosurgery, The 940th Hospital of Joint Logistics Support Force of PLA, Lanzhou, Gansu, China; 3 Department of Laboratory, The 940th Hospital of Joint Logistics Support Force of PLA, Lanzhou, Gansu, China

**Keywords:** appendicitis, Crohn's disease, gastrointestinal bleeding, hyperbaric oxygen therapy, hypobaric hypoxia, misdiagnosis, ultra-high altitude

## Abstract

This case describes a 31-year-old male who presented with acute exacerbation of pre-existing, undiagnosed Crohn's disease (CD) one week after rapid ascent to 4,500 meters. The patient had a long-standing history of recurrent oral ulcers and perianal fistulas. He was initially misdiagnosed with acute appendicitis based on right lower quadrant pain and fever, which resulted in emergency surgery. On postoperative day 9, life-threatening gastrointestinal bleeding (GIB) occurred. Management at the remote high-altitude facility was challenged by diagnostic limitations (lack of advanced imaging and endoscopic equipment), blood supply shortages, and lack of advanced interventions, which hindered the timely identification of the underlying disease and bleeding site. After emergency evacuation to lower altitude, GIB resolved spontaneously. Colonoscopy revealed ileocecal valve ulcers. Two-year follow-up with recurrent GIB and progressive intestinal ulceration further confirmed the diagnosis of CD. This case highlights ultra-high altitude exposure as a potential trigger for acute exacerbation of pre-existing CD, where atypical presentation often leads to misdiagnosis. The importance of recognizing extraintestinal manifestations in acute abdominal conditions is emphasized, alongside the critical challenges in managing complex cases in resource-limited high-altitude settings.

## Introduction

Crohn's disease (CD) is a chronic inflammatory bowel disorder with multifactorial and incompletely defined etiology, involving genetic susceptibility, immune dysregulation, gut microbiota imbalance, and environmental factors (1, 2). Recent evidence highlights environmental triggers, particularly high-altitude conditions characterized by hypoxia, hypobaria, and strong ultraviolet radiation, which may disrupt intestinal barrier function and immune homeostasis (3, 4). Chronic hypoxia can alter gut permeability, modulate microbiota, and provoke inflammatory responses in genetically predisposed individuals. Specifically, hypobaric hypoxia induces CD exacerbation via key pathways: upregulation of hypoxia-inducible factor (HIF)-1α impairs intestinal barrier function, disrupts pro-inflammatory (TNF-α, IL-6) and anti-inflammatory (IL-10) cytokine balance, and disturbs gut microbiota composition, collectively amplifying intestinal inflammation (5). CD often presents with diverse and non-specific clinical manifestations, leading to substantial challenges in early diagnosis. Extraintestinal manifestations (EIMs) such as recurrent oral ulcers and perianal lesions provide critical diagnostic clues but are frequently overlooked in clinical practice. When presenting as acute right lower quadrant pain, CD is frequently misdiagnosed as appendicitis (6–8). Here, we report a young male with a long-standing history of oral and perianal ulcers who developed acute exacerbation of pre-existing, undiagnosed CD following rapid ascent to 4,500 m, and was initially misdiagnosed with acute appendicitis. Postoperative life-threatening hemorrhage and delayed diagnosis underscore an association between ultra-high altitude exposure and CD exacerbation, and highlight diagnostic challenges in remote high-altitude settings. To the best of our knowledge, this represents the first case report describing acute exacerbation of pre-existing, undiagnosed CD induced by rapid ascent to 4,500 m, complicated by misdiagnosis as appendicitis, life-threatening postoperative bleeding, and resource-limited high-altitude management challenges. The uniqueness of this case lies in the combination of altitude-related CD exacerbation, surgical misdiagnosis, and spontaneous resolution of bleeding following evacuation to lower elevations. This case carries important clinical implications: it identifies ultra-high altitude as a potential trigger for CD exacerbation, highlights the diagnostic value of EIMs, and provides practical guidance for the management of complex CD cases in resource-limited high-altitude regions, thereby addressing an important gap in the current literature.

## Case description

**Figure 1:**
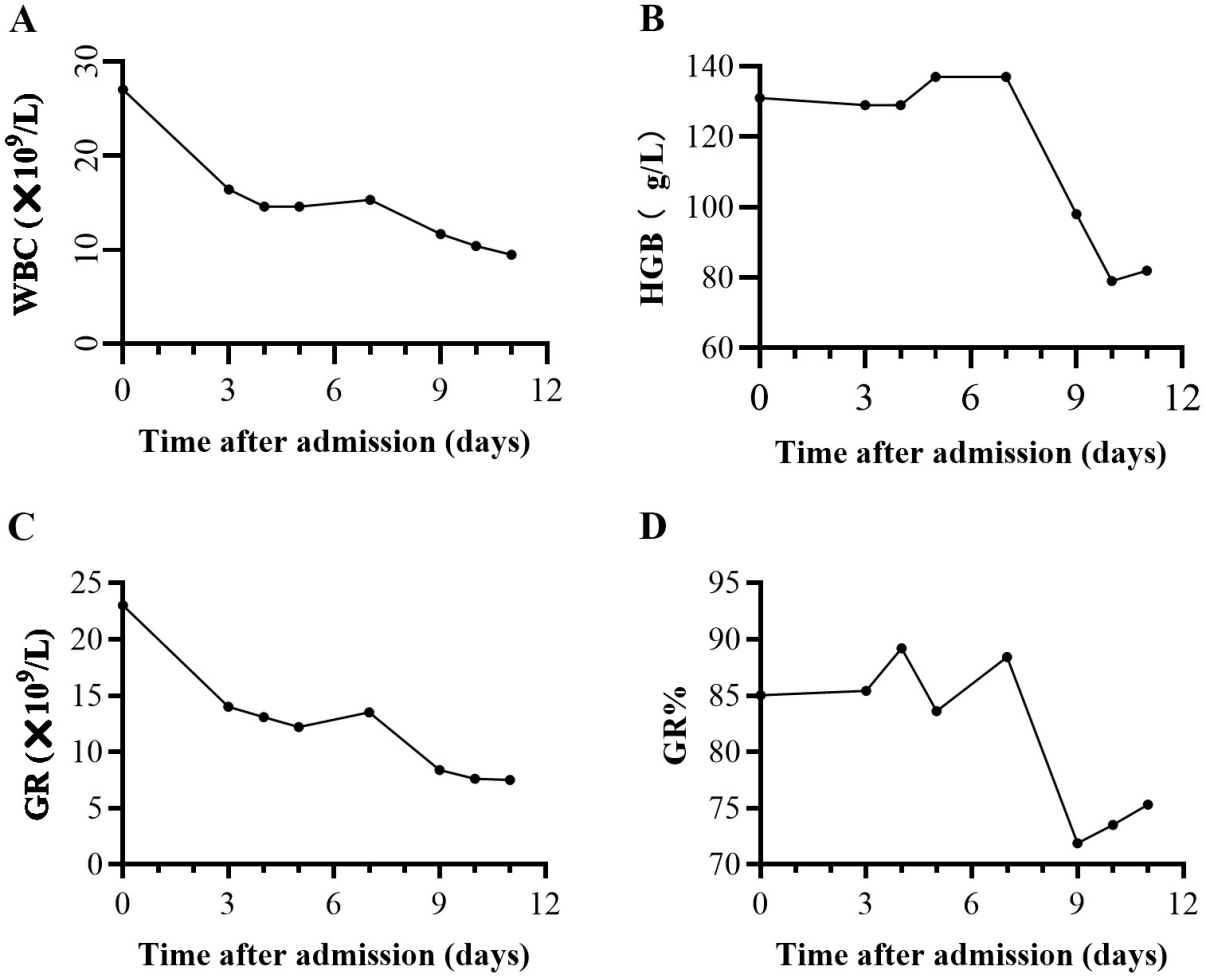
Laboratory test results of the patient after admission: the figures show the levels of WBC **(A)**, HGB **(B)**, GR **(C)**, and GR% **(D)**. WBC, white blood cell count; HGB, hemoglobin; GR, granulocyte count; GR%, granulocyte percentage.

**Figure 2:**
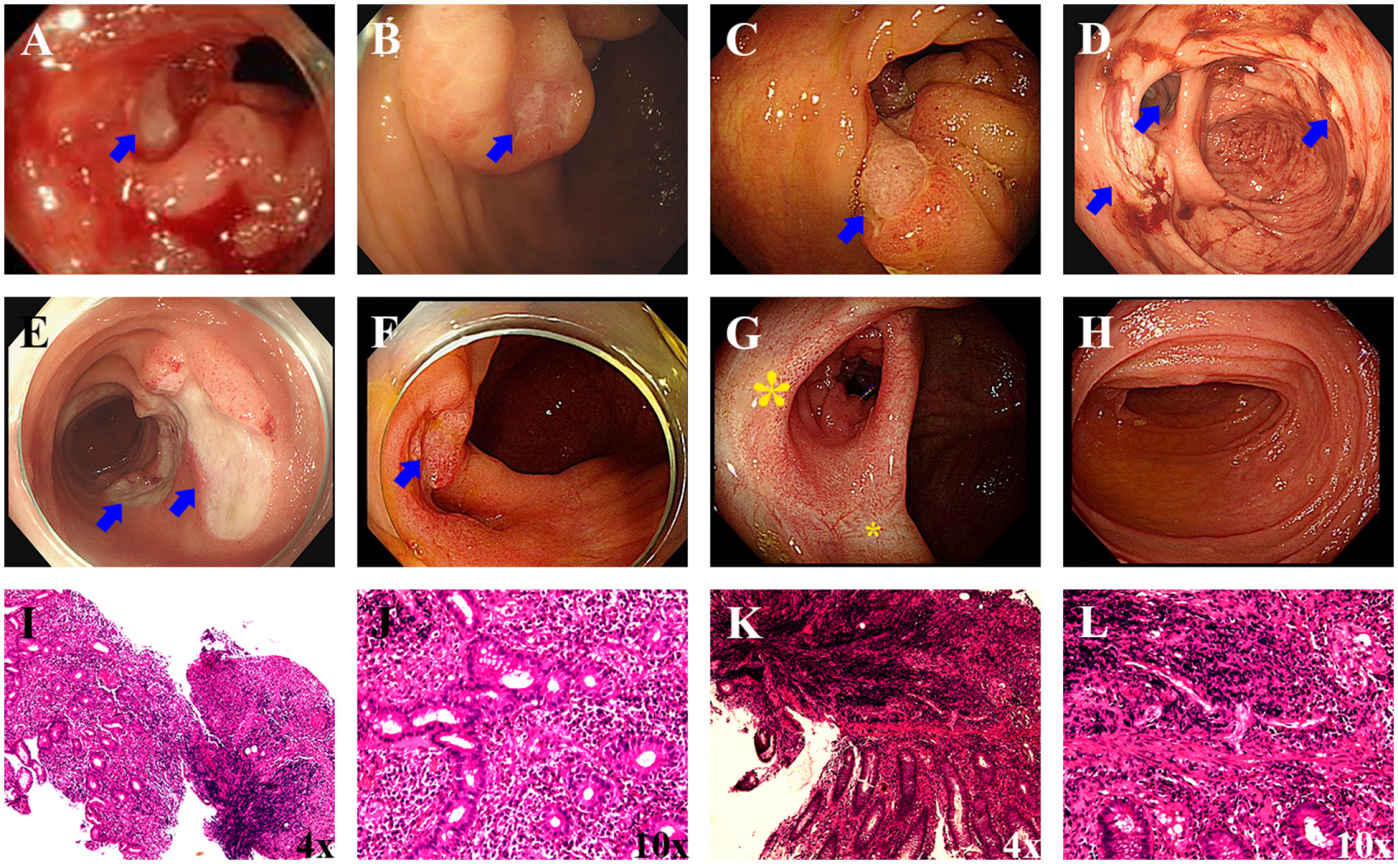
Dynamic evolution of colonoscopic findings and pathological characteristics in a Crohn's disease patient before and after infliximab (IFX) therapy. **(A)**: Pain-free colonoscopy performed on April 28, 2021 (22 days after the first episode of gastrointestinal bleeding) revealed a 0.6 cm × 0.8 cm ulcer at the inner orifice of the ileocecal valve, with surrounding mucosal congestion and edema (blue arrow). **(B)**: Follow-up pain-free colonoscopy on May 13, 2021 (37 days after the first episode of gastrointestinal bleeding) showed a persistent 0.6 cm × 0.6 cm ulcer at the ileocecal valve (blue arrow). **(C)**: The patient developed massive gastrointestinal bleeding on September 3, 2021; emergency pain-free colonoscopy demonstrated a 1.5 cm longitudinal ulcer on the cecal side of the ileocecal valve with visible microvascular stumps, and no obvious abnormalities were noted in other intestinal segments (blue arrow). **(D, E)**: The patient suffered another episode of massive gastrointestinal bleeding on September 26, 2023; emergency pain-free colonoscopy indicated multiple ulcers in the terminal ileum and ascending colon (blue arrow). **(F)**: Pain-free colonoscopy conducted on January 9, 2024, after 3 courses of IFX therapy showed only residual ulcer at the ileocecal valve, with no abnormalities detected in other intestinal segments (blue arrow). **(G, H)**: Follow-up pain-free colonoscopy on October 24, 2024, after 8 courses of IFX therapy revealed deformation of the ileocecal valve with visible white scars (yellow asterisk), and no ulcers were found in the terminal ileum and entire colon. **(I–L)**: Pathological examination of the lesion tissues collected from the terminal ileum **(I, J)** and ascending colon **(K, L)** after the massive gastrointestinal bleeding on September 26, 2023, indicated ulcers with acute active inflammatory changes and inflammatory granulomatous tissue hyperplasia.

A 31-year-old Han Chinese male was admitted to a medical clinic at an altitude of 4,500 meters on March 28, 2021. He had rapidly ascended to this high-altitude region one week prior to presentation. The patient presented with a 3-h history of migratory right lower abdominal pain and fever. Symptoms began as spontaneous periumbilical dull ache that progressed to sharp pain in the right lower quadrant, with a body temperature of 38.2 °C. No nausea, vomiting, or diarrhea was noted. Physical examination showed alert consciousness and marked tenderness and rebound tenderness at McBurney's point. Laboratory investigations showed a hemoglobin level of 131 g/L, a significantly elevated white blood cell count of 27.04 × 10^9^/L, and a neutrophil percentage of 85.1% (absolute count 23.01 × 10^9^/L). Other biochemical and coagulation parameters were unremarkable, consistent with an acute inflammatory response (Figure 1). Abdominal ultrasound demonstrated a thickened appendix measuring approximately 1.1 cm in diameter, highly suggestive of acute appendicitis. Based on these clinical and imaging findings, a working diagnosis of acute appendicitis was established, and the patient underwent emergency exploratory laparotomy and appendectomy under spinal anesthesia on the same day. Intraoperatively, the appendix showed no obvious swelling or purulent changes. Postoperatively, the patient was treated with ceftriaxone and ornidazole for anti-infective therapy and fluid resuscitation. Although fever resolved, intermittent abdominal pain persisted. On postoperative day 9 (April 6, 2021), the patient's condition deteriorated acutely, with massive melena accompanied by dizziness, palpitations, and pallor, indicating active gastrointestinal bleeding. Repeat laboratory testing showed a marked drop in hemoglobin from 131 g/L to 98 g/L. Immediate treatment included acid suppression, hemostatic therapy, and fluid resuscitation. Abdominal ultrasound showed no evidence of peritoneal effusion. On April 7, 2021, hemoglobin further decreased to 79 g/L (Figure 1B). Coagulation profiles revealed significantly reduced fibrinogen (0.81 g/L), elevated D-dimer (0.7 mg/L), prolonged thrombin time (22.8 s), prolonged prothrombin time (14.9 s), and prolonged activated partial thromboplastin time (38.8 s). The patient received an emergency transfusion of 4 units of packed red blood cells, with continued hemostatic and supportive care. However, dizziness and melena persisted. Under the medical conditions of the remote high-altitude area, the rescue efforts faced severe challenges: (1) Blood supply shortages: Local blood sources were scarce with limited reserves and difficult distribution, which hindered massive transfusion and compromised life-saving support.; (2) Insufficient diagnostic and therapeutic capabilities: The lack of equipment and technical conditions for emergency endoscopy and endoscopic hemostasis made it impossible to identify the bleeding site and implement effective interventions. Given the critical condition and the limitations of local treatment capabilities, an aeromedical evacuation procedure was immediately initiated to transfer the patient to a superior comprehensive hospital at a lower altitude. After arriving at the lower-altitude hospital, the patient's gastrointestinal bleeding improved significantly without specific hemostatic interventions, and hemoglobin levels gradually recovered. This phenomenon strongly suggested that leaving the high-altitude environment had a positive effect on alleviating the condition. After the patient's condition stabilized, gastroscopy, enteroscopy, and colonoscopy (extended imaging examinations) were performed, revealing active ulcers at the ileocecal valve (Figures 2A, B). Combined with the patient's 10-year long-standing history of recurrent oral ulcers, perianal abscesses, and fistulas—these were pre-existing manifestations of undiagnosed CD—the likelihood of inflammatory bowel disease (especially CD) was significantly elevated. During the subsequent two-year follow-up period, the patient was hospitalized multiple times due to recurrent gastrointestinal bleeding. Repeat colonoscopy showed an increase in the number and extent of intestinal ulcers, exhibiting the typical skip lesions and longitudinal ulcer characteristics of Crohn's disease (Figures 2C–F). Based on the patient's chronic disease course, typical extraintestinal manifestations (recurrent oral aphthous ulcers, perianal lesions), endoscopic features, and pathological examination results, a definitive diagnosis of Crohn's disease (A2L3B1 type, Montreal classification) was made (Figures 2I–L). The patient's condition stabilized after standardized treatment with biologics (infliximab) and other therapies (Figures 2G, H).

## Discussion

This case of high-altitude-induced acute exacerbation of CD highlights critical clinical presentation characteristics and their diagnostic implications, which are key to avoiding misdiagnosis. The patient's clinical manifestations were atypical: initial presentation with migratory abdominal pain mimicking acute appendicitis, accompanied by long-standing but overlooked extraintestinal manifestations (recurrent oral aphthous ulcers and perianal lesions), and subsequent life-threatening postoperative gastrointestinal bleeding. These presentations collectively contributed to the initial misdiagnosis, underscoring the importance of recognizing such atypical features in CD. Notably, recurrent oral aphthous ulcers and perianal lesions—typical extraintestinal manifestations of CD—often precede gastrointestinal symptoms by years (1). Systematic inquiry about these manifestations during initial evaluation can significantly improve diagnostic accuracy and avoid unnecessary surgical interventions. Particularly noteworthy is that perianal lesions hold important differential diagnostic value, helping to distinguish Crohn's disease from ulcerative colitis and other abdominal emergencies (2).

From an environmental perspective, this case provides strong evidence for an association between the ultra-high altitude environment and acute exacerbation of pre-existing, undiagnosed CD (9). The clear temporal relationship between the patient's rapid ascent to 4,500 meters and the onset of acute symptoms suggests that high-altitude exposure may have contributed to disease exacerbation, rather than directly inducing *de novo* CD. The underlying mechanisms involve multiple aspects: a hypoxic environment disrupts intestinal barrier function by downregulating tight junction proteins; HIF-mediated immune dysregulation promotes pro-inflammatory cytokine release; high-altitude also alters in gut microbiota, and sympathetic activation exacerbates intestinal ischemia (3, 6, 7). Particularly convincing is the spontaneous resolution of bleeding after the patient returned to lower altitude, which supports the role of altitude-related hypoxia in driving disease activity.

Given the critical role of altitude-induced hypoxia in triggering CD exacerbation in this case, hyperbaric oxygen therapy (HBOT) represents a promising supportive intervention for hypoxia-driven CD. HBOT improves tissue oxygenation, reverses hypoxia-induced cellular dysfunction, and thereby modulates intestinal inflammation—strengthening the mechanistic link between altitude-related hypoxia and CD activity. Recent studies confirm HBOT's efficacy in CD, particularly for refractory disease, perianal fistulas, and hypoxia-related inflammation. A prospective interventional study showed HBOT reduced C-reactive protein levels and Crohn's Disease Activity Index, ameliorated gut microbiota dysbiosis, and enhanced the efficacy of ustekinumab in refractory CD, with good safety profiles (10). A systematic review and meta-analysis of 16 studies demonstrated that HBOT achieved 89% clinical response and 59% remission in perianal fistulizing CD, with low adverse event rates (51.7 per 10,000 sessions) (11). By mitigating hypoxia-mediated inflammation and promoting mucosal healing, HBOT could serve as a targeted adjunctive therapy for CD patients with hypoxia-related exacerbation or refractory manifestations, especially in resource-limited high-altitude settings where immediate evacuation is not feasible.

This case carries important therapeutic implications for managing CD exacerbation induced by high-altitude exposure. First, early descent to lower-altitude areas is a critical intervention, as demonstrated by the spontaneous resolution of gastrointestinal bleeding in our patient after evacuation, which alleviates hypobaric hypoxia and halts disease progression. Second, timely high-flow oxygen supplementation can correct hypoxemia, reduce intestinal mucosal damage, and mitigate hypoxia-related inflammation. Third, once CD is confirmed, standardized therapy (e.g., biologics) should be initiated promptly to control chronic intestinal inflammation and prevent long-term recurrence, especially in patients with pre-existing undiagnosed CD at risk of altitude-induced flares. Collectively, these strategies provide practical clinical guidance for the management of similar cases in resource-limited high-altitude regions.

During the clinical course, surgical stress may have acted as an accelerator (12). The catastrophic bleeding on postoperative day 9 suggests that surgical intervention accelerated disease progression. Mechanistically, the systemic inflammatory response and increased intestinal permeability triggered by surgical trauma synergized with pre-existing high-altitude-induced mucosal damage through a “multiple-hit” pathway, ultimately leading to rapid disease deterioration.

From a clinical and healthcare system perspective, this case highlights three major challenges in remote high-altitude areas: limited diagnostic conditions, blood supply shortages, and inadequate treatment capabilities. To address these challenges and improve the management of similar cases, we propose integrated strategies combining clinical practice measures and system-level improvements. Clinically, four key steps should be prioritized: first, pre-ascension assessment for individuals with a history of extraintestinal manifestations (e.g., recurrent oral ulcers, perianal lesions) to screen for underlying CD and avoid altitude-induced exacerbation; second, early identification by clinicians in high-altitude regions, who should maintain a high index of suspicion for CD in patients with acute abdominal pain (especially those with extraintestinal manifestations) through detailed history taking and physical examination; third, optimized emergency management, where timely aeromedical evacuation to lower-altitude hospitals with advanced capabilities is life-saving for patients with severe complications (e.g., massive gastrointestinal bleeding); and fourth, consideration of HBOT as an adjunctive intervention for hypoxia-related CD exacerbation. At the system level, supporting measures include establishing a 5G-based telemedicine consultation system, conducting specialized training on inflammatory bowel disease recognition, building a regional blood supply network, and developing standardized aeromedical evacuation protocols. These integrated clinical and system strategies will effectively improve the comprehensive management for complex CD cases in remote high-altitude regions.

As a single case report, this study has limitations in generalizability, but it presents unique strengths in clarifying altitude-related CD exacerbation. Even though the proportion of individuals exposed to high altitudes is relatively low, this case provides valuable clinical insights for the diagnosis and treatment of CD in such settings.

## Conclusion

This case confirms that ultra-high altitude exposure may trigger or exacerbate Crohn's disease (CD), and surgical stress may further accelerate disease progression. For patients presenting at high altitudes with unexplained gastrointestinal symptoms, inflammatory bowel disease should be included in the differential diagnosis. Careful assessment of extraintestinal manifestations is essential for accurate and timely diagnosis. Moreover, strengthening medical infrastructure in remote high-altitude regions is critical for the effective management of complex cases.

## Data Availability

The original contributions presented in the study are included in the article/supplementary material, further inquiries can be directed to the corresponding authors.
